# *QuickStats*: Percentage* of Adults Aged ≥18 Years Who Felt That Crime Makes It Unsafe to Walk,^†^ by Sex and Age Group — National Health Interview Survey,^§^ United States, 2020

**DOI:** 10.15585/mmwr.mm7120a5

**Published:** 2022-05-20

**Figure:**
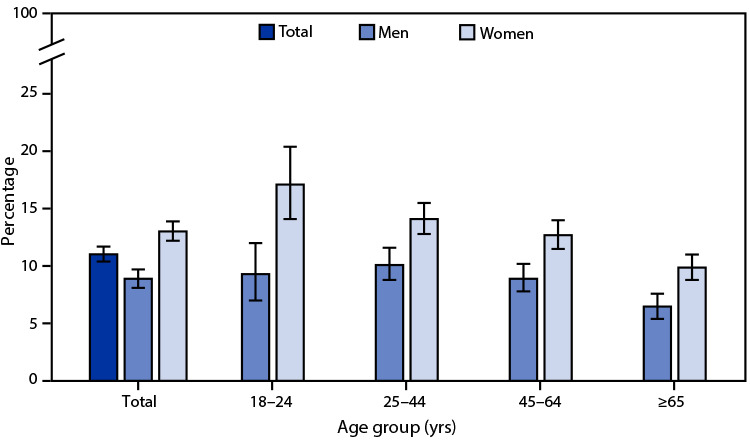
In 2020, 11.0% of adults aged ≥18 years felt that crime made it unsafe for them to walk. Percentages were lower for men (8.9%) than for women (13.0%). Men were less likely than women to feel unsafe walking because of crime in all age groups (18–24 years: 9.3% of men compared with 17.1% of women; 25–44 years: 10.1% of men compared with 14.1% of women; 45–64 years: 8.9% of men compared with 12.7% of women; ≥65 years: 6.5% of men compared with 9.9% of women). Among both sexes, adults aged ≥65 years were less likely to feel unsafe to walk than those in younger age groups.

**Source**: National Center for Health Statistics, National Health Interview Survey, 2020. https://www.cdc.gov/nchs/nhis.htm

For more information on this topic, CDC recommends the following link: https://www.cdc.gov/violenceprevention/index.html

